# Moderate-vigorous physical activity and health-related quality of life among Hispanic/Latino adults in the Hispanic Community Health Study/Study of Latinos (HCHS/SOL)

**DOI:** 10.1186/s41687-019-0129-y

**Published:** 2019-07-24

**Authors:** Priscilla M. Vásquez, Ramon A. Durazo-Arvizu, David X. Marquez, Maria Argos, Melissa Lamar, Angela Odoms-Young, Donghong Wu, Hector M. González, Wassim Tarraf, Daniela Sotres-Alvarez, Denise Vidot, Rosenda Murillo, Krista M. Perreira, Sheila F. Castañeda, Yasmin Mossavar-Rahmani, Jianwen Cai, Marc Gellman, Martha L. Daviglus

**Affiliations:** 1 Department of Neurosciences, University of California San Diego, 9500 Gilman Drive #0949, La Jolla, CA 92093-0949 USA; 2 Department of Preventive Health Sciences, Stritch School of Medicine, Loyola University Chicago, Maywood, IL USA; 3 Department of Kinesiology and Nutrition, University of Illinois at Chicago, Chicago, IL USA; 4 Division of Epidemiology and Biostatistics, School of Public Health, University of Illinois at Chicago, Chicago, IL USA; 5 Institute for Minority Health Research, University of Illinois at Chicago, Chicago, IL USA; 6 Department of Medicine, University of Illinois at Chicago, Chicago, IL USA; 7 Rush Alzheimer’s Disease Center and the Department of Behavioral Sciences, Rush University Medical Center, Chicago, IL USA; 8 Shiley-Marcos Alzheimer’s Disease Research Center, University of California San Diego, La Jolla, CA USA; 9 Institute of Gerontology and Department of Healthcare Sciences, Wayne State University, Detroit, MI USA; 10 Collaborative Studies Coordinating Center, Department of Biostatistics, University of North Carolina, Chapel Hill, NC USA; 11 School of Nursing and Health Sciences, University of Miami, Coral Gables, FL USA; 12 Psychological, Health, and Learning Sciences, University of Houston, Houston, TX USA; 13 Department of Social Medicine, University of North Carolina School of Medicine, Chapel Hill, NC USA; 14 Division of Health Promotion and Behavioral Science, Graduate School of Public Health, San Diego State University, San Diego, CA USA; 15 Department of Epidemiology & Population Health, Albert Einstein College of Medicine, Bronx, NY USA; 16 Department of Biostatistics, University of North Carolina, Chapel Hill, NC USA; 17 Behavioral Medicine Research Center, Miller School of Medicine, University of Miami, Miami, FL USA

**Keywords:** Hispanic, Latino, Physical activity, Accelerometer, Health-related quality of life, SF-12

## Abstract

**Background:**

Physical activity is a modifiable healthy behavior that has been shown to positively influence health-related quality of life. However, research examining the link between physical activity and health-related quality of life among Hispanic/Latino adults is limited and inconsistent. The purpose of this study is to assess whether accelerometer-measured moderate-vigorous physical activity (MVPA) is associated with self-reported (a) mental health-related quality of life, and (b) physical health-related quality of life among diverse Hispanic/Latino adults in the US.

**Methods:**

Cross-sectional data from 12,379 adults ages 18–74 years in 2008–2011, who participated in HCHS/SOL and had complete data were analyzed using complex survey design methods. Accelerometer data were categorized into no MVPA, low, moderate, and high MVPA. Health-related quality of life was assessed with the Short-Form 12 and we used the mental and physical component subscales where higher scores indicate better health-related quality of life. Multivariate linear regression models were used to derive adjusted means with 95% confidence intervals and linear trends.

**Results:**

We observed no significant linear trend between accelerometer-measured MVPA and mental health-related quality of life (p_trend_ = 0.73). There was a significant positive association between MVPA and physical health-related quality of life (p_trend_ < 0.001) where higher MVPA corresponded with higher scores in physical health-related quality of life. The adjusted means were 46.67 (44.85–48.48) for no MVPA, 49.33 (49.03–49.63) for low MVPA, 50.61 (50.09–51.13) for moderate MVPA, and 51.36 (50.86–51.86) for high MVPA.

**Conclusions:**

Among diverse Hispanic/Latino adults in the US, accelerometer-measured MVPA was associated with physical health-related quality of life, but not mental health-related quality of life. Future interventions should evaluate if increases in MVPA lead to improvements in health-related quality of life.

**Electronic supplementary material:**

The online version of this article (10.1186/s41687-019-0129-y) contains supplementary material, which is available to authorized users.

## Introduction

Health-related quality of life is defined as the impact of health on an individual’s well-being in the physical, mental, and social domains as well as their perceived ability to function [22]. Racial/ethnic disparities exist where some groups are disproportionately at risk of experiencing limitations and disabling conditions, which negatively influence quality of life. For example, research shows that Hispanics/Latinos tend to have lower self-reported quality of life than their non-Hispanic/Latino white and black counterparts [12]. Among older Hispanic/Latino adults the physical functioning component of health-related quality of life played a role in the association between frailty and mortality [25], and was associated with depressive symptoms [9]. Understanding how to maintain health-related quality of life and its mental and physical health components via health behaviors (e.g., physical activity) may inform strategies and recommendations to improve health and health-related quality of life.

Associations between accelerometer-measured moderate-vigorous physical activity (MVPA) and health-related quality of life have only recently been reported and findings show similar results as those observed with self-report physical activity (PA) data [4–6, 23]. There are different measures of health-related quality of life, and to our knowledge, no studies have examined the association between accelerometer-measured MVPA with mental health-related quality of life and physical health-related quality of life, which capture different aspects of quality of life as they relate to these components of health. Such an investigation may help to shed light on previous counter-intuitive results within the Hispanic/Latino population regarding the association between MVPA and health-related quality of life. More specifically, a study conducted with Hispanics/Latinos of Puerto Rican and Dominican backgrounds observed that older Hispanic/Latino adults engaged in more MVPA than their Chinese and African American counterparts as well as engaged in sufficient total MVPA per PA guideline recommendations, but had significantly worse total health-related quality of life [20]. The aim of the present study was to examine the association between accelerometer-measured MVPA and health-related quality of life among a diverse cohort of Hispanic/Latino adults. We hypothesize that greater accelerometer-measured MVPA will be associated with better physical and mental health-related quality of life. Addressing this gap can have implications for improving health-related quality of life via MVPA, which is a targetable and modifiable health behavior.

## Methods

### Study description

The Hispanic Community Health Study/Study of Latinos (HCHS/SOL) is a community-based cohort study, which aims to evaluate the health risks and protective factors of chronic conditions among a diverse sample of Hispanic/Latino adults in the United States (US). This study includes four US field centers (Bronx, New York; Chicago, Illinois; Miami, Florida; San Diego, California) and enrolled self-identified Hispanic/Latino adults (*N* = 16,415) of Puerto Rican, Mexican, Dominican, Cuban, Central American, and South American backgrounds between ages 18–74 years. Recruitment and baseline examination of participants took place between 2008 and 2011. Oversampling was implemented for adults aged 45–74 years [21]. Participants were ineligible if they were unable to physically travel to the field center, complete the study questionnaires, or if they were relocating residence in the subsequent 6 months [31]. Baseline examinations were standardized across all sites and were conducted in the preferred language of the participant (Spanish/English). Data on sociodemographic characteristics, clinic examinations, and questionnaire information were staff-administered. The Institutional Review Boards at each study site institution and coordinating center approved the study protocol. Informed consent was obtained for all participants at the beginning of the baseline examination visit. All measures used in the present study were collected at baseline examination. More detailed information about the study has been reported [21, 31], and additional study information may be found at http://www.cscc.unc.edu/hchs/.

### Measures

#### Physical activity

The Actical Accelerometer (model 198–0200-03; Minimiter Respironics, Bend, Oregon) recorded the frequency, duration, and intensity of PA during the time the accelerometer was worn. Participants were fitted with a belt and were instructed to wear the accelerometer for seven days at the hip, above the iliac crest and to only remove for swimming, showering, and sleeping [1]. Accelerometer data were processed with epoch length of 1 min and non-wear time was determined by using the Choi algorithm, which defined non-wear time as 90 consecutive minutes of zero counts, allowing only 1–2 min of nonzero counts within a 30-min window upstream and downstream of the 90-min timeframe [10]. Adherence to accelerometer-use was defined as having at least three days with a minimum of ten hours per day of data [26]. Adherence to accelerometer-use was higher among participants who were male, older, preferred Spanish over English, reported higher work activity or lower recreational activity, and had a lower body mass index. These findings and more detail are provided in a published article [13]. Given these differences, analyses in the present study were adjusted for missing accelerometer data and non-adherence to accelerometer-use using inverse probability weighting via a sampling weight derived by the HCHS/SOL Coordinating Center [13, 30].

We categorized accelerometer-measured MVPA into four levels: no MVPA, low, moderate, and high MVPA (minutes [min]/week). This variable was derived using the average of total moderate and/or vigorous minutes within a day across adherent days and then the average was multiplied by 7 (for each day of the week). The amount of moderate and vigorous minutes of PA were based on the 2008 PA guideline recommendations [18], specifically engaging in 150 min of moderate PA or 75 min of vigorous PA per week, or a combination of moderate and vigorous PA. No MVPA was defined as achieving less than 1 min of MVPA per week. Low MVPA was defined as less than 150 min of moderate PA or less than 75 min of vigorous PA. Moderate MVPA was defined as achieving between 150 and 300 min of moderate PA or between 75 and 150 min of vigorous PA. Lastly, high MVPA was defined as achieving more than 300 min of moderate PA or more than 150 min of vigorous PA (Additional file 1: Figure S1).

#### Health-related quality of life (short form (SF)-12 v.2): mental health-related quality of life & physical health-related quality of life

The SF-12 v.2 questionnaire is a standardized and widely used assessment of health-related quality of life, which captures eight health dimensions. This questionnaire has a total of twelve questions with each item weighted differently to yield the mental component score (MCS) and physical component score (PCS). The MCS captures the mental health-related quality of life and the health domains of vitality (i.e., energy), social functioning, mental health (psychological distress or psychological well-being), and limitations due to emotion problems (e.g., accomplishing less). The PCS captures the physical health-related quality of life and the health domains of physical functioning (e.g., climbing several flights), bodily pain, limitations due to physical functioning, and general health. The MCS and PCS were derived based on factor scores and general population norms developed for scoring the SF-12 v.2 [33]. The MCS and PCS are norm-based transformations of standardized z-scores of the constituent items which were scaled to a mean of 50 and standard deviation of 10 [33]. We assessed MCS and PCS continuously for this study, the total scores ranged from 0 to 100, and higher scores indicate better health-related quality of life.

##### Example questions for MCS include

During the past 4 weeks, how much of the time have you had any of the following problems with your work or other regular daily activities as a result of any emotional problems (such as feeling depressed or anxious)? With response options (i) all of the time (ii) most of the time (iii) some of the time (iv) a little of the time *or* (v) none of the time.

##### Example questions for PCS include

Does your health now limit you in these activities? (a) moderate activities, such as moving a table, pushing a vacuum cleaner, bowling or playing golf *and* (b) climbing several flights of stairs, with response options (i) yes, limited a lot (ii) yes, limited a little or (iii) no, not limited at all.

#### Covariates

The covariates used in this study were chosen based upon the literature describing the factors important to consider when evaluating Hispanic/Latino health and determined to be potential confounders of the association between MVPA and health-related quality of life. A personal information questionnaire was administered to collect demographic information. Age was measured continuously and sex was dichotomized to female or male. Marital status was categorized into single, married/living with partner, or separated/divorced/widower. Education was categorized into less than high school diploma or General Education Development certificate (GED), high school diploma or GED, and more than high school diploma or GED. Annual household income was categorized into less than $30,000 per year, between $30,000 - $50,000, more than $50,000, or not reported. Alcohol intake was categorized into three groups, those who never drank alcohol before, former drinkers, and current drinkers. Preferred language was determined by the language chosen for the baseline examination, and could be either Spanish or English. Place of birth was dichotomized to either born in a Latin American country or the US. The Latin American country category also includes participants born in Puerto Rico. Health insurance was dichotomized into having no health insurance or currently being insured. Study site was recorded for each participant and the HCHS/SOL field sites were the Bronx, New York; Chicago, Illinois; Miami, Florida; and San Diego, California. The covariate for Hispanic/Latino background was categorized into seven groups to include Central American, Cuban, Dominican, Mexican, Puerto Rican, South American, or more than one background group. In order to account for the role of some chronic conditions we also included the presence of cardiovascular disease, stroke/transient ischemic attack (TIA), liver disease, cancer, and inflammation or swelling of joints into the statistical models.

### Statistical analysis

**Fig. 1:**
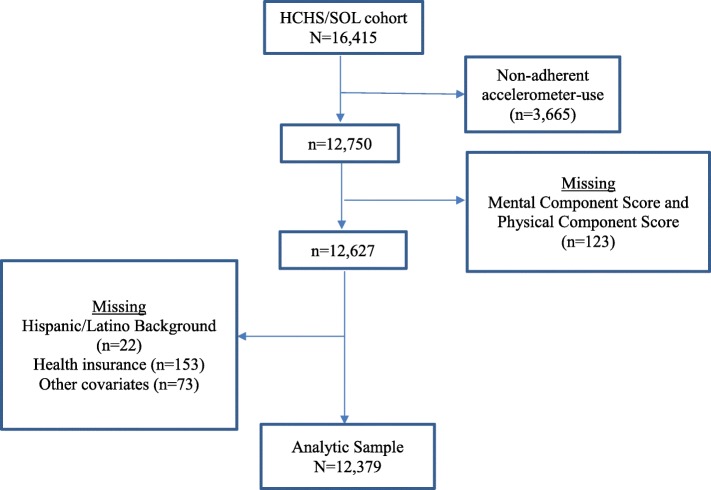
Consort diagram

The analytic sample had complete data on the study variables. Figure 1 provides the analytic sample consort diagram. Descriptive statistics for covariates and health-related quality of life were age-adjusted and reported by categories of accelerometer-measured MVPA. To derive the *p*-values for these bivariate associations we used the overall Wald test for continuous variables and the chi-square test for categorical variables. We used linear regression models to test the trend and examine the association between accelerometer-measured MVPA and the mental health-related quality of life and physical health-related quality of life, separately. The accelerometer-measured MVPA has been classified as four categories: no MVPA, low, moderate, and high MVPA. The adjusted means with 95% confidence intervals (CI) and p_trend_ were reported. We provide a supplemental table which presents estimates including covariates for the three linear models (Additional file 1: Tables S1 and S2). Analyses were conducted using a model to account for the various chronic conditions that might have influenced the association between MVPA and health-related quality of life. The model adjusted for age, sex, education, annual household income, study site, Hispanic/Latino background, marital status, alcohol intake, health insurance, preferred language, nativity, as well as cardiovascular disease, stroke, liver disease, cancer, and inflammation or swelling of joints. The adjusted means were adjusted to the overall mean values of the covariates. We conducted a sensitivity analysis using quartiles of MVPA and examined associations with MCS and PCS, and results were largely unchanged (results not shown).

All analyses accounted for the complex survey sampling design for HCHS/SOL including clustered and stratified sampling, and sampling weights to account for different probabilities of selection and non-response. Complex survey procedures in Stata Statistical Software, Release 14 (StataCorp LP, College Station, TX) were used for analyses.

## Results

### Descriptive statistics

The analytic sample included *N* = 12,379 participants from the HCHS/SOL cohort study. For MVPA, 1.8% were categorized to no MVPA, 55.9% in low MVPA, 23.2% in moderate MVPA, and 19.1% in high MVPA. Females were the majority in the no MVPA (62.8%) and low MVPA (61.3%) categories. Education ranged from 29.8% to 33.5% with less than a high school diploma across all categories of MVPA. A household income of less than $30,000 ranged from 40.2% to 50.4% across all categories of MVPA, and 67.1% to 77.8% preferred the Spanish language across all categories of MVPA. Overall, older Hispanic/Latino adults and Latinas/females were more concentrated in the lower MVPA category. The majority of those in the no MVPA category had current health insurance, while those in low MVPA had the lowest percentage of those with health insurance. Lastly, most in the no MVPA category were in Miami, FL, and most of the participants in the high MVPA category were in the Bronx, NY (Table 1).

**Table 1:** Age-adjusted characteristics for Hispanic/Latino adults by accelerometer-measured MVPA levels: HCHS/SOL

	Accelerometer-Measured MVPA Levels^b^				
	No MVPA (*n* = 240)	Low MVPA (*n* = 7325)	Moderate MVPA (*n* = 2752)	High MVPA (*n* = 2062)	*p*-value
All,^a^ %	1.8	55.9	23.2	19.1	0.004
Age in years, M (SE)	55.2 (3.1)	43.4 (0.3)	38.0 (0.4)	36.7 (0.4)	< 0.001
Female, N (%)	180 (62.8)	4966 (61.3)	1476 (45.5)	814 (33.0)	< 0.001
Marital Status, N (%)					
Single	40 (42.1)	1494 (30.3)	821 (37.1)	772 (41.2)	< 0.001
Married or Living with Partner	126 (38.8)	4173 (53.1)	1405 (46.5)	978 (43.6)	
Separated, Divorced, or Widower	74 (19.1)	1658 (16.6)	526 (16.4)	312 (15.2)	
Education, N (%)					
< High School/GED	119 (29.8)	2851 (31.2)	1059 (33.5)	762 (33.1)	0.21
High School/GED	44 (31.7)	1769 (27.8)	715 (28.1)	590 (29.5)	
> High School	77 (38.5)	2705 (41.0)	978 (38.4)	710 (37.5)	
Household Income, N (%)					
< $30,000	123 (50.4)	3256 (41.9)	1167 (40.2)	869 (42.9)	0.007
$30,000 - $50,000	67 (28.0)	2775 (37.2)	1077 (36.5)	811 (38.0)	
> $50,000	15 (6.3)	682 (11.3)	303 (13.8)	229 (11.4)	
Not Reported	35 (15.3)	612 (9.6)	205 (9.5)	153 (7.7)	
Alcohol Intake, N (%)					
Never	80 (30.4)	1621 (20.9)	430 (15.2)	258 (13.6)	< 0.001
Former	91 (25.3)	2444 (29.4)	916 (28.8)	680 (30.5)	
Current	69 (44.3)	3260 (49.6)	1406 (55.9)	1124 (55.8)	
Preferred Language, N (%)					
Spanish	205 (67.6)	6186 (77.8)	2194 (75.5)	1496 (67.1)	< 0.001
English	35 (32.4)	1139 (22.2)	558 (24.5)	566 (32.9)	
Place of Birth, N (%)					
Latin American-born	211 (76.4)	6267 (77.6)	2193 (75.4)	1517 (70.5)	< 0.001
US-born	29 (23.6)	1058 (22.4)	559 (24.6)	545 (29.5)	
Health Insurance, N(%)	166 (60.7)	3654 (47.4)	1438 (52.2)	1134 (56.7)	< 0.001
Study Site, N (%)					
Bronx	47 (22.5)	1388 (20.6)	825 (33.0)	783 (45.0)	< 0.001
Chicago	65 (17.3)	1920 (16.0)	746 (15.9)	566 (16.1)	
Miami	72 (37.8)	2043 (36.3)	458 (22.6)	266 (17.6)	
San Diego	56 (22.4)	1974 (27.1)	723 (28.5)	447 (21.4)	
Hispanic/Latino Background, N (%)					
Dominican	16 (7.3)	545 (8.1)	336 (12.3)	242 (12.3)	< 0.001
Central American	15 (6.0)	754 (7.5)	282 (7.6)	203 (7.0)	
Cuban	53 (29.0)	1233 (26.1)	228 (14.0)	120 (10.1)	
Mexican	86 (30.7)	3052 (37.3)	1180 (40.6)	835 (36.5)	
Puerto Rican	55 (18.4)	1085 (12.9)	438 (16.4)	456 (24.0)	
South American	12 (5.0)	491 (4.4)	197 (5.8)	124 (5.6)	
Other/More than one	3 (3.6)	165 (3.8)	91 (3.4)	82 (4.6)	
Health-Related Quality of Life, M (SE)					
Mental health-related quality of life	48.8 (1.5)	48.8 (0.3)	50.2 (0.3)	49.7 (0.4)	0.022
Physical health-related quality of life	45.1 (1.0)	49.6 (0.2)	50.7 (0.3)	50.8 (0.3)	< 0.001

Abbreviations: *M* Mean, *SE* Standard error

Unweighted N = 12,379

^a^Means (except age) and standard errors were weighted and adjusted for age. Proportions were weighted and adjusted for age. Frequencies are unweighted ^b^MVPA categories are defined as: No MVPA: ≥ 0 but < 1 min of moderate, and ≥ 0 but < 1 min of vigorous activity on average per week. Low MVPA: ≥ 1 but < 150 min of moderate or ≥ 1 but < 75 min of vigorous; or ≥ 1 but < 150 min of moderate/vigorous activity on average per week. Moderate MVPA: ≥ 150 min but < 300 min of moderate, or ≥ 75 min but < 150 of vigorous; or ≥ 150 but < 300 min of moderate/vigorous activity on average per week. High MVPA: ≥ 300 min of moderate, or ≥ 150 min of vigorous, or ≥ 300 min of moderate/vigorous activity on average per week [derived from total min per day with 1535–3961 counts per minute (cpm) for moderate activity, and ≥ 3962 cpm for vigorous activity; and ≥ 1535 cpm for moderate/vigorous activity- applies to all categories]

### Multivariable linear regression models

#### Mental health-related quality of life

We observed no significant linear trend (p_trend_ = 0.73) between accelerometer-measured MVPA and mental health-related quality of life (Table 2).

**Table 2:** Association between accelerometer-measured MVPA and health-related quality of life: HCHS/SOL

	No MVPA Adjusted^a^ Mean (95% CI)	Low MVPA Adjusted Mean (95% CI)	Moderate MVPA Adjusted Mean (95% CI)	High MVPA Adjusted Mean (95% CI)	p_trend_
Mental health-related quality of life	50.23 (46.91–53.54)	49.08 (48.56–49.59)	49.85 (49.23–50.46)	49.16 (48.37–49.95)	0.73
Physical health-related quality of life	46.67 (44.85–48.48)	49.33 (49.03–49.63)	50.61 (50.09–51.13)	51.36 (50.86–51.86)	< 0.001

Note: p_trend_ is the *p* value for MVPA added as a continuous covariate in linear regression- See Additional file 1

^a^Adjusted for age, sex, education, household income, study site, and Hispanic/Latino background, marital status, alcohol intake, health insurance, preferred language, place of birth, and cardiovascular disease, stroke, liver disease, inflammation or swelling of joints, and cancer

MVPA categories are defined as: No MVPA: ≥ 0 but < 1 min of moderate, and ≥ 0 but < 1 min of vigorous activity on average per week. Low MVPA: ≥ 1 but < 150 min of moderate or ≥ 1 but < 75 min of vigorous; or ≥ 1 but < 150 min of moderate/vigorous activity on average per week. Moderate MVPA: ≥ 150 min but < 300 min of moderate, or ≥ 75 min but < 150 of vigorous; or ≥ 150 but < 300 min of moderate/vigorous activity on average per week. High MVPA: ≥ 300 min of moderate, or ≥ 150 min of vigorous, or ≥ 300 min of moderate/vigorous activity on average per week [derived from total min per day with 1535–3961 counts per minute (cpm) for moderate activity, and ≥ 3962 cpm for vigorous activity; and ≥ 1535 cpm for moderate/vigorous activity- applies to all categories]

#### Physical health-related quality of life

There was a significant positive linear trend between accelerometer-measured MVPA and physical health-related quality of life. The adjusted means and 95% CI were 46.67 (44.85–48.48) for no MVPA, 49.33 (49.03–49.63) for low MVPA, 50.61 (50.09–51.13) for moderate MVPA, and 51.36 (50.86–51.86) for high MVPA (p_trend_ < 0.001) (Table 2).

## Discussion

We found that accelerometer-measured MVPA was associated with self-reported physical health-related quality of life, but not with mental health-related quality of life. Our hypotheses were partially supported by our findings. Health-related quality of life is multidimensional and captures the impact of health on quality of life, which is not captured by direct measures of population health, life expectancy, and causes of death [8, 27]. MVPA is an established healthy behavior that prevents poor health outcomes, and existing evidence has shown associations between MVPA and health-related quality of life [3, 5] However, limitations include the lack of generalizability to underrepresented groups, namely, Hispanics/Latinos, the use of data from mostly older adults, and the lack of observational data. The present study extends previous research by adding to the benefits of MVPA, which includes better physical health-related quality of life among Hispanic/Latino adults.

To our knowledge, few studies have evaluated the association between accelerometer-measured MVPA and health-related quality of life, but our findings align with findings from previous studies. Research has shown that accelerometer-measured MVPA was independently and positively associated with the physical health-related quality of life, but not mental health-related quality of life of older adults [34]. A longitudinal study demonstrated that engaging in PA was linked with the improved physical health-related quality of life component [2], and another study showed that individuals who engaged in MVPA had better reported health-related quality of life [32]. The studies however did not include Hispanic/Latino adults in their sample. As such, the findings of our study provide evidence that even in the event of different profiles of MVPA among diverse Hispanic/Latino adults [24] and within a younger population, higher accelerometer-measured MVPA is associated with better physical health-related quality of life. If the associations hold longitudinally then there is potential to improve health-related quality of life among Hispanic/Latino adults via increasing accelerometer-measured MVPA. The aligning of our findings with previous research with older adults has possible implications for healthy aging, given that associations may be observed earlier in adulthood and continue with aging. The lack of association of accelerometer-measured MVPA with mental health-related quality of life component may be a result of the construct being limited to a broad conceptualization of psychological distress or psychological well-being, and there may be other aspects of mental health that we are unable to explore in this study.

The linear association we were able to detect in our study demonstrated that higher MVPA corresponds with the higher physical health-related quality of life component. This physical health component captures physical limitations and, by extension, aspects of disability. A study using the National Health Interview Survey (NHIS), a nationally representative sample of US adults, observed that 21.4% of Hispanic/Latino adults had a disability or physical limitations [11]. In addition, higher rates of disability or physical limitations were observed among Puerto Ricans compared to African Americans, whereas Cubans had lower rates than non-Hispanic/Latino whites. While the causes of disability are wide ranging, disability or physical limitations due to health conditions may be prevented by engaging in MVPA [7, 28]. Our study is cross-sectional and it could be that those with better health-related quality of life engage in more MVPA. However, previous studies have shown the positive impact of engaging in MVPA on physical health as well as the detrimental influence of low amounts of MVPA [15]. A study among community-dwelling older adults observed an improvement in physical function as a result of PA [19], and another study demonstrated that components of physical frailty, such as difficulty with walking and balance in addition to psychological and social frailty, predicted future scores of quality of life [16]. Higher MVPA is linked to higher physical health-related quality of life in the present study, which is salient in light of the high prevalence of disability and limitations due to physical functioning among Hispanic/Latino adults.

Overall, Hispanic/Latino adults in our study had lower physical health-related quality of life compared to nationally representative values for non-institutionalized US adults [17]. For Hispanic/Latino adults there may be many factors that play a role in health-related quality of life [14], and strategies that mitigate the negative influences are warranted. Accessibility to spaces that promote MVPA and addressing barriers to becoming physical active are plausible options to help address health-related quality of life and physical health among Hispanic/Latino adults. The notion of higher MVPA positively influencing health-related quality of life and physical health-related quality of life may also be extended to other comorbid conditions, such as cardiovascular risk factors. Researchers have estimated a small change in physical health-related quality of life can be clinically significant. For instance, an approximate 3- to 5-unit change in the physical health-related quality of life can be clinically significant for hypertension [29]. The importance of health-related quality of life has been established given the link to clinical health outcomes and mortality. Strategies to improve health-related quality of life, however are less understood and less established. In our study, we provide initial evidence on one possible approach which is to engage in higher MVPA, we found that even small increments in MVPA per our MVPA categories were associated with better physical health-related quality of life. We observed the nearly 3-point higher score in physical health-related quality of life among those with low MVPA compared to no MVPA, this is promising given barriers to achieving high MVPA. Lastly, those engaging in higher MVPA, compared to no MVPA had a nearly 5-point higher score in physical health-related quality of life. Engaging in MVPA has benefits for health and specific chronic conditions, and our study adds to existing literature demonstrating the benefits on health-related quality of life. This provides cross-sectional evidence on possible improvements in health-related quality of life with increasing MVPA. Our findings are, however, subject to reverse causation where those with higher health-related quality of life had the capability of engaging in higher MVPA. Prospective studies are needed to examine these associations longitudinally.

Limitations of this study include the cross-sectional analyses, such that we cannot establish causality and the possibility of reverse causation. We did not stratify results by Hispanic/Latino background or sex, and associations may differ based on these demographics. We also did not assess these associations by specific chronic conditions or diseases, although we did adjust for many in our final model. This study examined the association of MVPA such that associations may be different when examining light-intensity PA as well as water-based physical activity, such as swimming, and health-related quality of life. Light-intensity PA may be especially more relevant for older adults, however this was not examined in the present study. Lastly, the health-related quality of life measured may be subject to reporting bias given that it was self-reported data.

## Conclusion

This study evaluated the association between accelerometer-measured MVPA and health-related quality of life, specifically mental health-related quality of life and physical health-related quality of life among Hispanic/Latino adults. We observed a significant association between accelerometer-measured MVPA and physical health-related quality of life, but not mental health-related quality of life. Our findings contribute to existing research using accelerometer-measured MVPA as well as to evaluating this association among diverse Hispanic/Latino adults in the US. The findings of this study provide evidence to continue to address barriers to engaging in MVPA for Hispanic/Latino communities. In addition to the benefits of MVPA on overall health, our study suggests the extension of benefits to health-related quality of life. The findings in this study can inform and be incorporated into programs aiming to increase MVPA, improving health, and health-related quality of life.

## Additional file

Additional file 1: **Table S1.** Mental Health-Related Quality of Life: Estimated Regression Coefficients. **Table S2.** Physical Health-Related Quality of Life: Estimated Regression Coefficients. **Figure S1.** MVPA Categories. (DOCX 121 kb)

## Data Availability

Please refer to the study website further information, http://www.cscc.unc.edu/hchs/. Restrictions apply to the availability of these data, which were used under license for the current study, and so are not publicly available.

## Abbreviations

CI: Confidence intervals
GED: General Education Development certificate
HCHS/SOL: Hispanic Community Health Study/Study of Latinos
MCS: Mental Component Score
MVPA: Moderate-vigorous physical activity
PA: Physical activity
PCS: Physical Component Score
SF-12: Short Form-12
US: United States
