# Supplementary material for: Moderate-vigorous physical activity and health-related quality of life among Hispanic/Latino adults in the Hispanic Community Health Study/Study of Latinos (HCHS/SOL)
Source: J Patient Rep Outcomes. 2019 Jul 24;3:45. doi: 10.1186/s41687-019-0129-y (PMC6656822; doi:10.1186/s41687-019-0129-y)
Supplement: Supplementary file 1 — Table S1. Mental Health-Related Quality of Life: Estimated Regression Coefficients. Table S2. Physical Health-Related Quality of Life: Estimated Regression Coefficients. Figure S1. MVPA Categories. (DOCX 121 kb) [file 41687_2019_129_MOESM1_ESM.docx]

Additional file 1

Table S1. Mental Health-Related Quality of Life: Estimated Regression Coefficients

| Parameter | Estimate | Standard Error | t Value | p_trend_ |
| --- | --- | --- | --- | --- |
| **Intercept** | 34.0654835 | 14.9583051 | 2.28 | **0.0231** |
| **MVPA** | 0.0841021 | 0.2471580 | 0.34 | 0.7338 |
| **Age** | 0.0208270 | 0.0154412 | 1.35 | 0.1779 |
| **Female** | -4.4857684 | 0.3509665 | -12.78 | **<.0001** |
| **Education** | | | | |
| High School | 0.8434270 | 0.4661886 | 1.81 | 0.0709 |
| >High School | 1.5218253 | 0.4340994 | 3.51 | **0.0005** |
| **Annual Household Income** | | | | |
| $30,000-$50,000 | 2.7816719 | 0.4208288 | 6.61 | **<.0001** |
| >$50,000 | 4.0614392 | 0.5983186 | 6.79 | **<.0001** |
| Missing | 2.4923742 | 0.6132266 | 4.06 | **<.0001** |
| **Study Site** | | | | |
| Chicago, IL | 0.3532105 | 0.6834423 | 0.52 | 0.6055 |
| Miami, FL | 1.4626777 | 0.6859181 | 2.13 | **0.0334** |
| San Diego, CA | -0.0685568 | 0.7626196 | -0.09 | 0.9284 |
| **Hispanic/Latino Background** | | | | |
| Cuban | -1.0118703 | 0.8356625 | -1.21 | 0.2264 |
| Puerto Rican | -1.9966470 | 0.8042982 | -2.48 | **0.0133** |
| Dominican | 0.4517670 | 0.9455393 | 0.48 | 0.6330 |
| Central American | 0.1822015 | 0.6966461 | 0.26 | 0.7938 |
| South American | -0.7657274 | 0.7782149 | -0.98 | 0.3255 |
| More than one | -1.6922847 | 1.1655660 | -1.45 | 0.1470 |
| **Marital Status** | | | | |
| Married | 0.2843234 | 0.5048305 | 0.56 | 0.5735 |
| Separated | 0.1346609 | 0.5297905 | 0.25 | 0.7994 |
| **Alcohol Intake** | | | | |
| Former | -0.8375503 | 0.4531570 | -1.85 | **0.0650** |
| Current | -1.4652628 | 0.4473472 | -3.28 | **0.0011** |
| **No Health Insurance** | -0.0147414 | 0.3369367 | -0.04 | 0.9651 |
| **Spanish Preferred Language** | -0.1462836 | 0.5552745 | -0.26 | 0.7923 |
| **2^nd^ Generation** | -0.8533607 | 0.6102645 | -1.40 | 0.1625 |
| **Chronic Conditions** | | | | |
| Swelling of Joints | -3.0198422 | 0.6915665 | -4.37 | **<.0001** |
| Liver Disease | -1.4706351 | 0.6557711 | -2.24 | **0.0253** |
| Cancer | 0.6545595 | 1.1514557 | 0.57 | 0.5699 |
| Stroke/TIA | -1.1364249 | 1.4848442 | -0.77 | 0.4443 |
| Angina | -2.6486178 | 0.9462714 | -2.80 | **0.0053** |
| **Number of Accelerometer Wear Days** | 2.6443341 | 2.4676352 | 1.07 | 0.2843 |

**Table S2. Physical Health-Related Quality of Life: Estimated Regression Coefficients**

| Parameter | Estimate | Standard Error | t Value | p_trend_ |
| --- | --- | --- | --- | --- |
| **Intercept** | 41.0166056 | 4.81374154 | 8.52 | **<.0001** |
| **MVPA** | 1.1214265 | 0.13941068 | 8.04 | **<.0001** |
| **Age** | -0.1239800 | 0.01032994 | -12.00 | **<.0001** |
| **Female** | 0.1022652 | 0.23838187 | 0.43 | 0.6681 |
| **Education** | | | | |
| High School | 0.8717527 | 0.31173523 | 2.80 | **0.0053** |
| >High School | 1.6497590 | 0.29769463 | 5.54 | **<.0001** |
| **Annual Household Income** | | | | |
| $30,000-$50,000 | 1.2754248 | 0.28165208 | 4.53 | **<.0001** |
| >$50,000 | 2.5388078 | 0.38870972 | 6.53 | **<.0001** |
| Missing | 0.9723622 | 0.37426641 | 2.60 | **0.0096** |
| **Study Site** | | | | |
| Chicago, IL | 2.3102701 | 0.38429659 | 6.01 | **<.0001** |
| Miami, FL | 3.8265307 | 0.47357434 | 8.08 | **<.0001** |
| San Diego, CA | 2.5788248 | 0.43279813 | 5.96 | **<.0001** |
| **Hispanic/Latino Background** | | | | |
| Cuban | -0.0005610 | 0.58392973 | -0.00 | 0.9992 |
| Puerto Rican | -0.2887029 | 0.44726322 | -0.65 | 0.5188 |
| Dominican | 0.9342643 | 0.57797847 | 1.62 | 0.1065 |
| Central American | -0.5630098 | 0.50291298 | -1.12 | 0.2633 |
| South American | 0.0851834 | 0.52683210 | 0.16 | 0.8716 |
| More than one | 0.1023599 | 0.61581984 | 0.17 | 0.8680 |
| **Marital Status** | | | | |
| Married | 0.4268355 | 0.26628474 | 1.60 | 0.1094 |
| Separated | -0.1435363 | 0.52014978 | -0.28 | 0.7827 |
| **Alcohol Intake** | | | | |
| Former | 0.0355016 | 0.33234170 | 0.11 | 0.9150 |
| Current | 0.9580112 | 0.31524774 | 3.04 | **0.0025** |
| **No Health Insurance** | -0.9924950 | 0.25574226 | -3.88 | **0.0001** |
| **Spanish Preferred Language** | 0.1667202 | 0.40345176 | 0.41 | 0.6796 |
| **2^nd^ Generation** | -0.9661960 | 0.51187760 | -1.89 | **0.0595** |
| **Chronic Conditions** | | | | |
| Swelling of Joints | -6.7617984 | 0.40425970 | -16.73 | **<.0001** |
| Liver Disease | -1.8577548 | 0.41596910 | -4.47 | **<.0001** |
| Cancer | -2.6995868 | 0.59936655 | -4.50 | **<.0001** |
| Stroke/TIA | -2.7598712 | 0.80052926 | -3.45 | **0.0006** |
| Angina | -2.8521446 | 0.42859204 | -6.65 | **<.0001** |
| **Number of Accelerometer Wear Days** | 1.4059874 | 0.79370276 | 1.77 | 0.0770 |

**Figure S1. MVPA Categories**


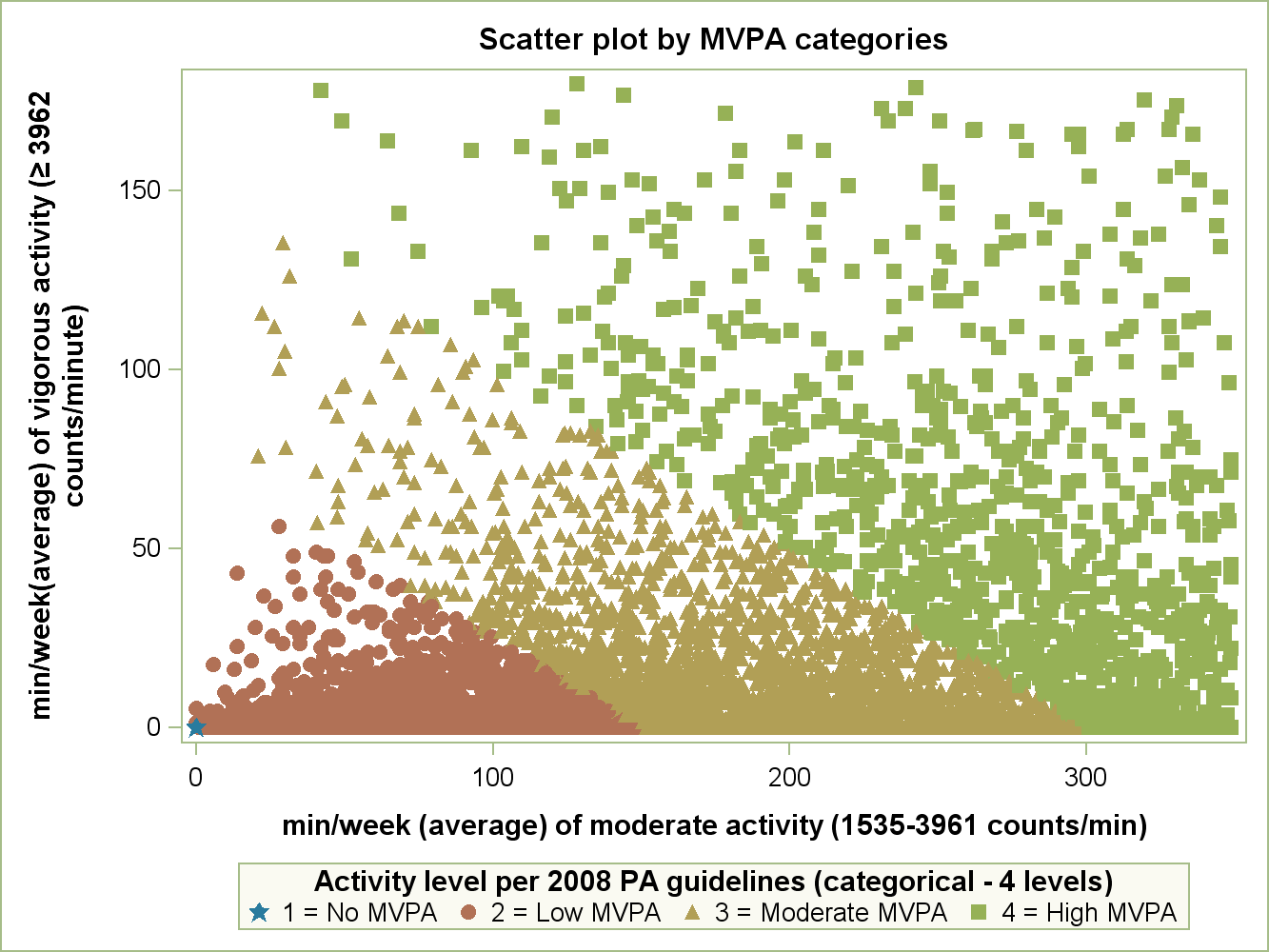


Please note that the scatterplot does not account for the sampling weights for our dataset.
